# Investigating the effects of statins on ischemic heart disease allowing for effects on body mass index: a Mendelian randomization study

**DOI:** 10.1038/s41598-022-07344-8

**Published:** 2022-03-03

**Authors:** Shun Li, C. M. Schooling

**Affiliations:** 1 School of Public Health, Li Ka Shing Faculty of Medicine, The University of Hong Kong, 7 Sassoon Rd, Pokfulam, Hong Kong, China; 2 School of Public Health and Health Policy, The City University of New York, 55 W 125 St, New York, NY 10027 USA

**Keywords:** Genetic association study, Cardiovascular diseases

## Abstract

Despite effective lipid reduction and corresponding benefits for cardiovascular disease prevention and treatment, statins have pleiotropic effects potentially increasing the risk of ischemic heart disease (IHD), particularly by increasing body mass index (BMI). We assessed whether the effects of genetically mimicked statins on IHD were strengthened by adjusting for BMI in men and women. We also assessed if increasing BMI was specific to statins in comparison to other major lipid-lowering treatments in current use, i.e., proprotein convertase subtilisin/kexin type 9 (PCSK9) inhibitors and ezetimibe. Using univariable and multivariable Mendelian randomization (MR) we found genetically mimicked effects of statins increased BMI (0.33, 95% confidence interval (CI) 0.28 to 0.38), but genetically mimicked PCSK9 inhibitors and ezetimibe did not. Genetically mimicked effects of statins on IHD reduction in both sexes (odds ratio (OR) 0.55 per unit decrease in effect size of low-density lipoprotein cholesterol (LDL-c), 95% confidence interval (CI) 0.40 to 0.76), was largely similar after adjusting for BMI, in both men (OR 0.48, 95% CI 0.38 to 0.61) and women (OR 0.66, 95% CI 0.53 to 0.82). Compared with variations in *PCSK9* and *NPC1L1*, only variation in *HMGCR* was associated with higher BMI. The effects on IHD of mimicking statins were similar after adjusting for BMI in both men and women. The BMI increase due to statins does not seem to be a concern as regards the protective effects of statins on IHD, however other factors driving BMI and the protective effects of statins could be.

## Introduction

Statins reduce low-density lipoprotein cholesterol (LDL-c) by inhibiting 3-hydroxy-3-methylglutaryl–coenzyme A reductase (HMGCR), giving a corresponding reduction in morbidity and mortality from cardiovascular disease (CVD)^1,2^. Despite effective lipid modification, pleiotropic effects, possibly increasing the risk of CVD^3,4^ have also been noticed during long-term use of statins. Specifically, statin treatment may increase risk of type 2 diabetes^5^ and is associated with higher body mass index (BMI)^3,4^, both of which increase CVD risk^6,7^. Effects on adiposity and glucose metabolism may influence the choice of lipid-lowering treatment^3^ and statin adherence^8^.

Genetically predicted proprotein convertase subtilisin/kexin type 9 (PCSK9) inhibitors, ezetimibe and statins similarly reduce the risk of cardiovascular events per unit reduction in LDL-c^5^, but only statins are thought to increase BMI. Given statins increase BMI, this suggests greater effects of statins than other lipid modifiers per unit change in LDL-c. More importantly, whether these associations manifest differently by sex remains unknown, although differences by sex are evident for some effects of statins^9^. Sex-specific information may contribute to understanding the pleiotropic effects of statins and inform their use, particularly for prevention where the risks and benefits can be finely balanced, and differ by sex^10^.

We used Mendelian randomization (MR) to obtain unconfounded sex-specific estimates of the effects of genetically mimicked statins on IHD allowing for their effects on BMI using the largest available sex-specific genome-wide association studies (GWAS). Specifically, for IHD and LDL-c we used GWAS of the UK Biobank cohort study provided by Neale lab (http://www.nealelab.is/uk-biobank/) and for BMI a GWAS of the UK Biobank and the Genetic Investigation of ANthropometric Traits (GIANT) consortium^11^. To identify if the BMI increase was unique to statins, we also sex-specifically assessed whether this effect was evident for the other two common lipid modifiers, PCSK9 inhibitors and ezetimibe. Finally, we replicated the analysis using genetic predictors of LDL-c and genetic associations with IHD from other GWAS.

## Results

**Figure 1:**
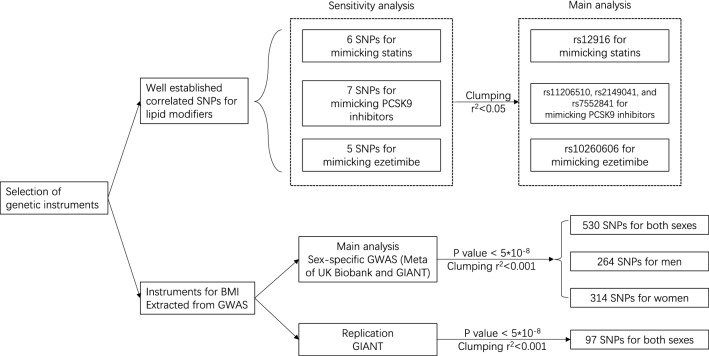
Selection of genetic instruments for lipid modifiers and BMI.

We used a single nucleotide polymorphisms (SNP) rs12916 to mimic statins. In sensitivity analysis, the six correlated SNPs from *HMGCR* (Supplementary Table S1-2, r^2^ > 0.13) were used taking account of their correlations. Of the 7 SNPs mimicking effects of PCSK9 inhibitors from *PCSK9* (Supplementary Table S1 and Supplementary Table S3), the three independent (r^2^ < 0.05) SNPs (rs11206510, rs2149041 and rs7552841) were used in the main analysis (Fig. 1). Of the 5 correlated SNPs mimicking effects of ezetimibe from *NPC1L1* (Supplementary Table S1 and Supplementary Table S4, r^2^ > 0.05), rs10260606 (proxy of rs2073547, r^2^ = 0.99) was used in the main analysis given it had the strongest association with LDL-c (Fig. 1). These associations with LDL-c for the SNPs mimicking statins, PCSK9 inhibitors and ezetimibe are given overall (Supplementary Table S5), in men (Supplementary Table S6) and in women (Supplementary Table S7).

### Instrument strength

The F statistic for most SNPs used to genetically mimic the effects of statins, PCSK9 inhibitors and ezetimibe were > 10 for men, women and overall (Supplementary Table S5-7). The F statistics for the 264 SNPs (men), 314 SNPs (women) and 530 SNPs (overall) predicting BMI were all greater than 10, with median 49.8, 42.2 and 41.8 respectively.

### Associations of genetically mimicked lipid modifiers with BMI

**Figure 2:**
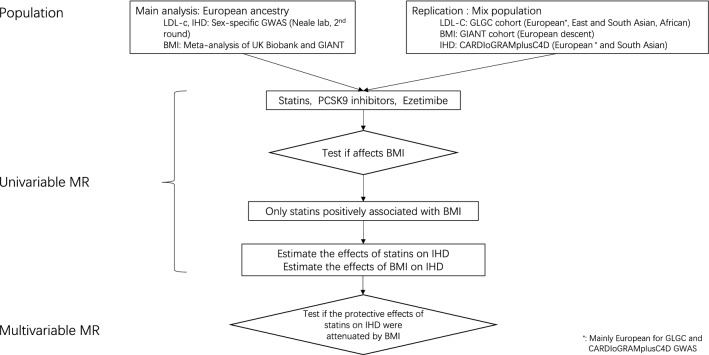
Flowchart of the analyses and populations used in the study.

Genetically mimicked effects of statins increased BMI in men and women whereas genetically mimicked effects of PCSK9 inhibitors and of ezetimibe were not associated with BMI (Fig. 2). Further analysis for PCSK9 inhibitors and ezetimibe was not conducted, given their lack of association with BMI.

### Associations of genetically mimicked effects of statins with BMI and IHD

Genetically mimicked effects of statins reduced the risk of IHD overall (odds ratio (OR) 0.55, 95% CI 0.40 to 0.76), and in men and women (Table 1). Genetically predicted BMI was positively associated with IHD for both sexes, with a slightly greater effect in women (OR 1.65, 95% CI 1.45 to 1.89) than in men (OR 1.40, 95% CI 1.26 to 1.54). However, MR-Egger intercepts for BMI suggested pleiotropic effects for women (*P*_intercept_ = 0.005), where the MR-Egger estimate was much smaller than the IVW estimate (Table 1). However, the I_GX_2 was low (less than 90%; 89.8% overall, 85.0%, 84.9% for men and women, respectively), suggesting the violation of the NOME assumption for the MR-Egger estimates which were biased towards the null^12^, and that the MR-Egger estimates may not be reliable for one-sample MR^13^. The weighted median, consistent between one-sample and two-sample MR^13^, showed positive associations of BMI with IHD in both sexes (Table 1).

**Table 1:** Univariable MR for genetically mimicked statins and BMI on IHD.

Exposure	Sex	SNP	Method	OR	95% CI	*P* value	*P*_intercept_
Statins	Overall	1	IVW	0.55	0.40 to 0.76	2.83*10^–4^	
		6	IVW	0.56	0.39 to 0.79	1.01*10^–3^	
	Men	1	IVW	0.52	0.33 to 0.82	4.92*10^–3^	
		6	IVW	0.56	0.31 to 1.00	0.050	
	Women	1	IVW	0.53	0.31 to 0.90	0.019	
		6	IVW	0.51	0.30 to 0.89	0.018	
BMI	Overall	530*	IVW	1.57	1.46 to 1.70	5.14*10^–31^	
		530*	MR-Egger	1.36	1.12 to 1.66	0.002	0.123
		530*	MR-Median	1.52	1.37 to 1.68	1.50*10^–15^	
	Men	264	IVW	1.40	1.26 to 1.54	4.14*10^–11^	
		264	MR-Egger	1.31	1.00 to 1.71	0.052	0.610
		264	MR-Median	1.29	1.13 to 1.46	1.07*10^–4^	
	Women	314	IVW	1.65	1.45 to 1.89	2.47*10^–13^	
		314	MR-Egger	1.01	0.70 to 1.46	0.949	0.005
		314	MR-Median	1.45	1.19 to 1.75	1.78*10^–4^	

In multivariable MR the conditional F-statistics for effects of genetically mimicked statins were 10.8 overall, 5.7 for men and 11.9 for women and for BMI were 61.8 overall, 31.6 for men and 56.6 for women. The multivariable MR-Egger intercept was significant overall and for men (*P* = 0.002, 2.33*10^–8^, respectively) and the corresponding modified Q statistic was also significant (877.5 and 435.3, respectively; *P* values < 0.001), so we focused on the MR-Egger estimates overall (OR 0.60, 95% CI 0.52 to 0.69) and for men (OR 0.48, 95% CI 0.38 to 0.61). The Q statistic (399.6, *P* < 0.001) also suggested pleiotropic effects for women, althought the MR-Egger intercept was not significant, where the MR-Egger estimate was also used (OR 0.66, 95% CI 0.53 to 0.82).

**Figure 3:**
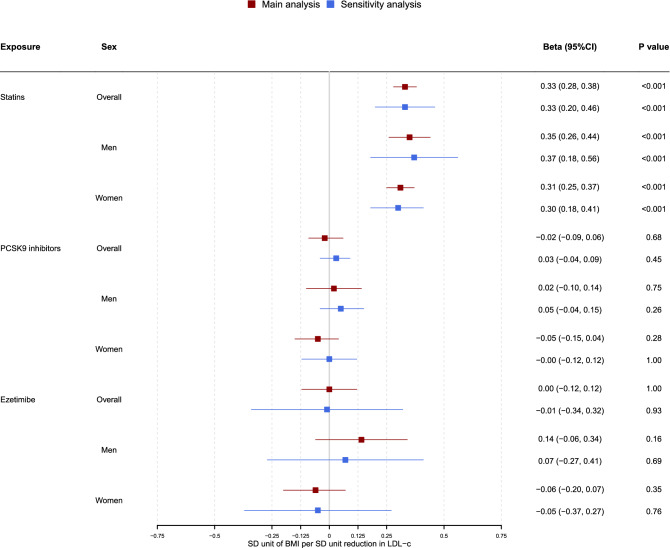
Forest plot for univariable MR estimates comparing the effects of genetically mimicked statins, PCSK9 inhibitors and ezetimibe on BMI. The red colour represents the main analysis using independent SNPs and the blue colour represents the sensitivity analysis using correlated SNPs. Assuming the UK Biobank standard deviations for LDL-c (0.871 mmol/L), BMI (4.77 kg/m^2^) and height (1.70 m), for each one mmol/L lower LDL-c from statin use for overall (Beta _statin-BMI_ = 0.33), BMI increases by 1.81 kg/m^2^, and weight increases by 5.2 kg.

**Figure 4:**
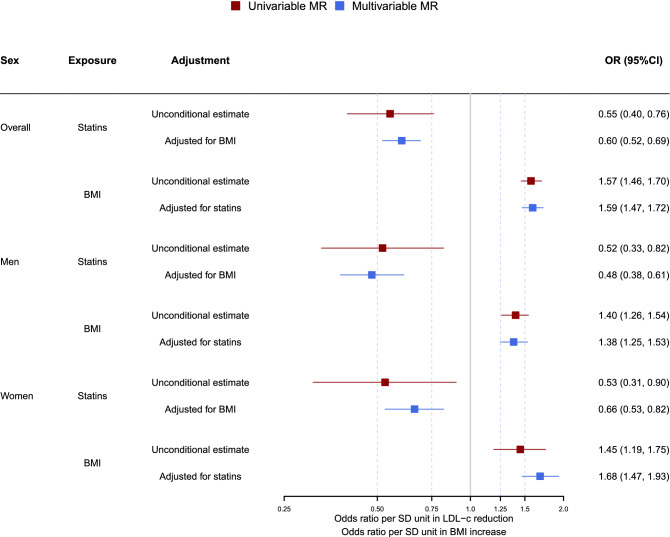
Forest plot for main analysis comparing the univariable MR estimates with the multivariable MR estimates for the effects of genetically mimicked statins and BMI on IHD. In the main analysis, the lead SNP, rs12916 was used for mimicking statins. The MR-Egger method was used for presenting the univariable MR estimates, given that the multivariable MR-Egger estimates were suggested for overall, men and women.

Compared with the univariable estimates for genetically mimicked effects of statins on IHD, the multivariable estimates (OR) adjusted for BMI were slightly smaller for men using MR-Egger, and were slightly larger for women and overall using MR-Egger (Table 2 and Fig. 4). Sensitivity analysis including correlated SNPs showed similar estimates as above for both univariable and multivariable MR (Figs. 3, 4 and Tables 1, 2).

**Table 2:** Multivariable MR of statin and BMI on IHD.

Sex	Exposure	SNP	IVW			MR-Egger			
			OR	95% CI	*P* value	OR	95% CI	*P* value	*P*_intercept_
Overall	Statin	1	0.65	0.57 to 0.75	1.30*10^–6^	0.60	0.52 to 0.69	4.41*10^–12^	0.002
	BMI	530*	1.62	1.5 to 1.75	2.38*10^–33^	1.59	1.47 to 1.72	4.66*10^–32^	
Overall	Statin	6	0.65	0.57 to 0.74	8.78*10^–7^				
	BMI	530*	1.62	1.50 to 1.75	8.76*10^–34^				
Men	Statin	1	0.66	0.53 to 0.82	0.019	0.48	0.38 to 0.61	1.07*10^–9^	2.33*10^–8^
	BMI	263*	1.46	1.32 to 1.61	1.88*10^–12^	1.38	1.25 to 1.53	1.03*10^–10^	
Men	Statin	6	0.67	0.54 to 0.82	0.020				
	BMI	263*	1.46	1.32 to 1.62	1.00*10^–12^				
Women	Statin	1	0.67	0.54 to 0.82	8.66*10^–4^	0.66	0.53 to 0.82	2.58*10^–4^	0.755
	BMI	313*	1.68	1.47 to 1.92	2.71*10^–9^	1.68	1.47 to 1.93	3.70*10^–14^	
Women	Statin	6	0.66	0.54 to 0.81	4.82*10^–4^				
	BMI	313*	1.68	1.47 to 1.92	2.72*10^–9^				

*Excluded one SNP that were in LD (r^2^ > 0.001) with rs12916: rs2119753, rs2588785 and rs214249 for overall, men and women, respectively.

### Replication

We replicated the primary results using a different GWAS, i.e., GLGC for LDL-c, UK Biobank and GIANT^14^ seperately for BMI and CARDIoGRAMplusC4D for IHD which had fewer SNPs for BMI. Similarly, genetically mimicked effects of statins increased BMI, whereas these effects were not evident for PCSK9 inhibitors or ezetimibe (Table 3). Genetically mimicked effects of statins were negatively associated with IHD, while BMI were positively associated with IHD (Table 4). Compared with the univariable estimates, the multivariable estimates (OR) for effects of statins on IHD adjusting for BMI were largely similar (Table 5).

**Table 3:** Univariable MR for genetically mimicking statins, PCSK9 inhibitors, ezetimibe on BMI using IVW (GLGC for LDL-c, GIANT from MR-base for BMI).

Exposure	SNP	Beta	95% CI	*P* value
Statins	1	0.25	0.16 to 0.33	4.33*10^–9^
	6	0.28	0.15 to 0.41	4.18*10^–5^
PCSK9 inhibitors	3	-0.05	-0.12 to 0.03	0.22
	7	-0.02	-0.10 to 0.05	0.52
Ezetimibe	1*	0.05	-0.12 to 0.22	0.56
	5	0.09	-0.15 to 0.32	0.46

*rs2073547 was available and therefore was used as the main SNP for ezetimibe.

**Table 4:** Univariable MR for genetically mimicking statins and BMI on IHD (CARDIoGRAMplusC4D for IHD, GLGC for LDL-c, GIANT from MR-base for BMI).

Exposure	SNP	Method	OR	95% CI	*P* value	*P*_intercept_
Statins	1	IVW	0.61	0.48 to 0.79	1.59*10^–4^	
	6	IVW	0.66	0.51 to 0.84	8.66*10^–4^	
BMI	93*	IVW	1.44	1.25 to 1.65	2.30*10^–7^	
	93*	MR-Egger	1.69	1.14 to 2.50	0.009	0.402

*Excluded 3 palindromic SNPs (rs1558902, rs17001654, rs9641123) and rs12016871 that was not available in the CARDIoGRAMplusC4D.

**Table 5:** Multivariable MR of statins and BMI on IHD (CARDIoGRAMplusC4D for IHD, GLGC for LDL-c, GIANT from MR-base for BMI).

Analysis	Exposure	SNP	IVW			MR-Egger			
			OR	95% CI	*P* value	OR	95% CI	*P* value	*P*_intercept_
1	Statin	1	0.58	0.48 to 0.70	1.36*10^–9^	0.62	0.51 to 0.76	2.78*10^–6^	0.136
	BMI	88*	1.46	1.30 to 1.68	1.76*10^–9^	1.46	1.28 to 1.65	7.85*10^–9^	
2	Statin	6	0.58	0.49 to 0.69	4.72*10^–10^				
	BMI	88*	1.46	1.29 to 1.65	1.15*10^–9^				

*Excluded extra 5 SNPs that were in LD (r^2^ > 0.01) with rs12916: rs11583200, rs2033529, rs2112347, rs2650492, rs4787491.

## Discussion

Consistent with previous findings our study provides genetic evidence that statins raise BMI^3,4^, while PCSK9 inhibitors or ezetimibe do not affect BMI^3,15^. BMI increases the risk of IHD in both sexes^16,17^. Our study adds by showing that the BMI increase caused by statins may not detract from the protective effects of mimicking statins on IHD in both men and women.

Although statins are known to increase BMI, the underlying mechanism remains unknown. Previous studies have shown dietary changes, particularly higher caloric intake for statin users^18^. Compared with statins, the other two lipid modifiers, PCSK9 inhibitors and ezetimibe however, did not affect BMI, which implies that higher BMI is not a consequence of lipid lowering, particularly of LDL-c lowering. Differences in effects on BMI for the three lipid modifiers may be related to their mechanisms of action. PCSK9 inhibitors enhance the clearance of LDL-c by increasing LDL receptors^19^, while ezetimibe reduces the cholesterol absorption^20^. In contrast, statins inhibit cholesterol synthesis^21^, an upstream bioprocess involving de novo synthesis in Leydig cells, which affects steroidogenesis^22,23^. Genetic scores for *HMGCR*, *PCSK9* and *NPC1L1* have been similarly associated with a slightly increased risk of diabetes^24^, and LDL-c lowering genetic variants located around genes targeted by lipid-lowering therapy have been positively associated with risk of type 2 diabetes^25^. Taken together these studies indicate detrimental pleiotropic effects of these variants on diabetes as a possible consequence of lipid lowering, which is different from our findings, suggesting statins specifically increase BMI, possibly indicating a different underlying pathway.

Although the estimates for BMI on IHD were not entirely consistent, it is likely that BMI increases the risk of IHD, with possibly more convincing estimates for men than women, which may also be partially explained by the hormone changes resulting from higher BMI. BMI raises plasma insulin in both sexes^26^, resulting in testosterone stimulation *in vivo*^27^, possibly via gonadotropin releasing hormone^28^. Testosterone stimulation is more relevant in men than in women given that men have higher testosterone than women^29^. Meanwhile, testosterone increases the risk of IHD more in men than women^9^. An MR study has also shown insulin raises the risk of CVD in men but not in women^30^. Conversely, BMI reduces testosterone in men^31^. As such, the overall effects of statins mediated by BMI in men may be relatively neutral due to these pleiotropic effects.

The effects of genetically mimicking statins on IHD were largely similar after adjusting for BMI in both men and women, suggesting that BMI may not necessarily mediate or undermine the effects of statins on IHD, despite the positive association of BMI with IHD. BMI is not a well-defined intervention reflecting measurable factors, such as diet and exercise, where different ways of changing BMI may have different effects^32^. Thus, a BMI increase due to statin therapy, but may not necessarily lead to IHD. However, although the association of statins with IHD does not seem to be mediated by BMI, the increase of BMI per 1 mmol/L LDL-c corresponds to an increased risk of IHD of OR 1.13 95% CI 1.07 to 1.19. Statins also increase the risk of type 2 diabetes, however, we did not adjust for type 2 diabetes because type 2 diabetes may be a downstream effect of BMI, whereby higher BMI increases the risk of type 2 diabetes^33^, likely resulting in higher risk of IHD^6^. Similarly, we did not adjust for HbA1c which may also partially mediate the effects of BMI on IHD^34^.

Despite providing sex-specific estimates and showing sex differences for the direct effects of statins on IHD allowing for BMI, this study has some limitations. First, instruments should satisfy three key assumptions, i.e., the instrument is strongly associated with the exposure, not related to factors confounding the exposure-outcome relation, and influences the outcome only via effects on the exposure. The genetic variants used are well established mimics of statins, PCSK9 inhibitors and ezetimibe^24^. However, despite the high conditional F-statistics for BMI in the multivariable MR, the conditional F-statistics for genetically mimicked statins were low, and the Q statistic was high for overall estimate and men in the multivariable MR, suggesting pleiotropic effects for these two analyses, which were addressed by using multivariable MR-Egger. Pleiotropic effects for multivariable MR were not evident for women in this study. We cannot exclude the possibility that the SNPs mimicking effects of statins influence IHD through other pathways given the potential pleiotropic effects of these SNPs^35^.

Our findings, mainly based on populations of European descent, may not be transportable to other populations. Nevertheless, causes are consistent across populations, although relevance of the causal mechanism may vary by population^36^. Third, we replicated the results based on other large GWAS consortia for both exposure and outcome. However, due to lack of availability of sex-specific summary statistics for IHD in relevant populations, this study lacks sex-specific replication. Fourth, we assumed a linear relation for the association of lipid modifiers and BMI with IHD^37^. Fifth, linear regression was used for a binary outcome in the GWAS of IHD from Neale lab, which may generate false positives particularly when case numbers are small or minor allele frequency (MAF) is low (http://www.nealelab.is/blog/2017/9/11/details-and-considerations-of-the-uk-biobank-gwas). However, the number of IHD cases was large and all SNP MAFs were over 1%. Sixth, our exposure statin use is similar to a dichotomized binary exposure, which does not have a clear interpretation^38^. However, statin use was instrumented by a number of SNPs and our estimates may correspond to different levels of statin use. Seventh, the estimates here do not necessarily reflect the exact risk reduction from pharmacologic treatments^39^, but the lifelong effects of endogenous exposures per unit decrease in LDL-c estimated by Mendelian Randomization^9^, which are usually greater than the short-term effects of pharmacologic intervention from an RCT^40^.

## Methods

### Genetic predictors mimicking effects of lipid modifiers

We used well-established genetic variants mimicking effects of statins (rs12916, rs10066707, rs17238484, rs2006760, rs2303152 and rs5909), PCSK9 inhibitors (rs11206510, rs2149041, rs7552841, rs2479409, rs2479394, rs10888897 and rs562556) and ezetimibe (rs10260606 (proxy of rs2073547, r^2^ = 0.99), rs217386, rs7791240, rs10234070 and rs2300414)^24^, from the corresponding genes encoding the target protein for each lipid modifier (*HMGCR*, *PCSK9* and *NPC1L1*, respectively). As the few SNPs mimicking each lipid modifier are correlated, independent (r^2^ < 0.05) genetic predictors of the lipid modifiers strongly associated with LDL-c (p < 5*10^–8^) were used in the primary analysis, i.e., rs12916 for statins, rs11206510, rs2149041, rs7552841 for PCSK9 inhibitors and rs10260606 for ezetimbe. For sensitivity analysis all correlated SNPs given above for each lipid modifier were used^24^ with a correlation matrix between SNPs for Europeans obtained from the 1000 Genomes catalogue using LDlink (https://ldlink.nci.nih.gov). MR estimates are presented in effect sizes of LDL-c reduction to mimic the effects of statins, PCSK9 inhibitors and ezetimibe, extracted from LDL-c genetic summary statistics from the UK Biobank where a sex-specific GWAS is available (http://www.nealelab.is/uk-biobank). The summary statistics are based on white British people and were adjusted for age, age^2^, sex, age*sex, age^2^*sex and the first 20 principal components for both sexes, and were adjusted for the first 20 principal components, age and age^2^ for sex-specific GWAS. For replication, the associations of the relevant SNPs with LDL-c were also taken from the Global Lipids Genetics Consortium (GLGC), a meta-analysis of 188 577 participants mainly of European descent adjusted for age and sex, and excluding people known to be on lipid lowering medication^41^ (Fig. 2).

### Genetic predictors of BMI

SNPs that were strongly (p < 5*10^–8^) and independently (r^2^ < 0.001) associated with BMI were also obtained from the largest sex-specific GWAS of the meta-analysis^11^ of the UK Biobank GWAS and the GIANT consortium (Fig. 1), which included 806,834 participants of European ancestry, including 374,756 men and 434,794 women adjusted for age, sex, recruitment centre, genotyping batches and 10 principal components. GIANT^14^ with 339,224 participants of European ancestry was adjusted for age, age^2^, and any study specific covariates, such as principal components. In GIANT estimates for unrelated individuals were stratified by sex and case/control status, while for family based studies sex was included as a covariate.

### Genetic associations with IHD

Sex-specific associations of selected SNPs with IHD were also extracted from a UK Biobank GWAS provided by Neale lab, which concerned up to 361,194 participants with 20,857 cases of IHD, including 167,020 men with 15,056 cases of IHD and 194,174 women with 5,801 cases of IHD. Sex-combined associations with IHD were from the UK Biobank were taken from SAIGE genetic summary statistics^42^. Replication was based on CARDIoGRAMplusC4D (Coronary ARtery DIsease Genome wide Replication and Meta-analysis (CARDIoGRAM) plus The Coronary Artery Disease (C4D) Genetics) consortium, a meta-analysis of GWAS of IHD mainly based on people of European descent including 60,801 cases and 123,504 controls adjusted for study specific covariates and over-dispersion^43^ (Fig. 2).

### Statistical analysis

Instrument strength was assessed by the F statistic using an established approximation, i.e., the square of beta of the SNP-exposure association divided by the square of its standard error^12^.

Genetic associations for each SNP were aligned on the same effect allele for exposure and outcome. Inverse-variance weighting (IVW) with multiplicative random effects^44^ combining SNP-specific Wald estimates (SNP-outcome divided by SNP-exposure) was used to obtain overall and sex-specific estimates of the associations of genetically predicted effects of lipid modifiers (i.e., statins, PCSK9 inhibitors and ezetimibe) with BMI and IHD, and estimates of the associations of genetically predicted BMI with IHD.

Multivariable MR was used to assess overall and sex-specific associations of genetically predicted effects of lipid modifiers with IHD adjusted for BMI. We combined the genetic variants for lipid modifiers and BMI in the multivariable MR, removed any BMI variants correlated with statins SNPs (r^2^ < 0.001) and extracted their associations with LDL-c, BMI and IHD using corresponding GWAS. We used the conditional F-statistic to give an estimate of instrument strength conditional on the other exposure in multivariable MR^45^. We also used the modified Q statistic to assess horizontal pleiotropic effects using the WSpiller/MVMR package^45^, and used the MR-Egger estimate when a significant Q statistic^46,47^ suggested violation of exclusion restriction assumption.

### Sensitivity analysis

In sensitivity analysis, we used all SNPs mimicking effects of statins, PCSK9 inhibitors and ezetimibe using correlation matrices obtained from LDlink (https://ldlink.nci.nih.gov/). We used MR-Egger as sensitivity analysis for the univariable analysis given its less stringent assumptions assuming the instrument strength is independent of the direct effect^48^, while IVW assumes balanced pleiotropy. However, we did not use MR-Egger for correlated instruments due to concerns about interpretability^47^. We used the IGX2, an adaptation of the I^2^ statistic, to assess the violation of the no measurement error (NOME) assumption, where a low IGX2 (< 90%) suggests a violation of the assumption for MR-Egger^12^. We also used the weighted median as it provides valid estimates when more than 50% of the information comes from valid instruments^49^.

All statistical analyses were conducted using R (version 4.0.3; R Foundation for Statistical Computing, Vienna, Austria). The univariable and multivariable MR estimates were obtained using the MendelianRandomization R package and the WSpiller/MVMR package to obtain the conditional F-statistic and the modified Q statistic.

### Ethics approval and consent for publication

Ethics approval was not required as only publicly available data was used in this study.

## Conclusion

Compared with variations in *PCSK9* and *NPC1L1*, *HMGCR* was uniquely associated with higher BMI for both sexes. Similar positive associations of BMI with IHD were found for men and women. The effects of mimicking statins on IHD per unit decrease in LDL-c were similar after adjusting for the harmful effects of BMI in both men and women.

## Supplementary Information

Supplementary Information 1.

Supplementary Information 2.

## Data Availability

The datasets generated and analysed during the current study are available in the UK Biobank repository, (http://www.nealelab.is/uk-biobank).

## Abbreviations

BMI: Body mass index
CARDIoGRAMplusC4D: Coronary artery disease genome wide replication and meta-analysis plus the coronary artery disease genetics
CVD: Cardiovascular disease
GIANT: Genetic investigation of anthropometric traits
GLGC: Global lipids genetics consortium
GWAS: Genome-wide association studies
HbA1c: Glycated haemoglobin
IHD: Ischaemic heart disease
IVW: Inverse-variance weighting
LD: Linkage disequilibrium
LDL: Low-density lipoprotein
MR: Mendelian randomization
OR: Odds ratio
PCSK9: Proprotein convertase subtilisin/kexin type 9
SD: Standard deviation
SNP: Single nucleotide polymorphisms
