# Supplementary material for: Investigating the effects of statins on ischemic heart disease allowing for effects on body mass index: a Mendelian randomization study
Source: Sci Rep. 2022 Mar 3;12:3478. doi: 10.1038/s41598-022-07344-8 (PMC8894423; doi:10.1038/s41598-022-07344-8)
Supplement: Supplementary file 2 — Supplementary Information 2. [file 41598_2022_7344_MOESM2_ESM.docx]

**Supplementary table legends**

**Table S1.** Established genetic variants in the HMGCR, PCSK9 and NCP1L1 gene that mimic statins, PCSK9 inhibitors and ezetimibe respectively. **Table S2.** Correlation matrix for genetic variants mimicking statins (effect allele for each SNP was aligned as the effect allele shown at Table S1). **Table S3.** Correlation matrix for genetic variants mimicking PCSK9 inhibitors (effect allele for each SNP was aligned as the effect allele shown shown at Table S1). **Table S4.** Correlation matrix for genetic variants mimicking ezetimibe (effect allele for each SNP was aligned as the effect allele shown shown at Table S1). **Table S5.** The associations of SNPs included with exposures and outcome for overall. **Table S6.** The associations of SNPs included with exposures and outcome for men. **Table S7.** The associations of SNPs included with exposures and outcome for women
